# Platelet Transfusion—Insights from Current Practice to Future Development

**DOI:** 10.3390/jcm10091990

**Published:** 2021-05-06

**Authors:** Annina Capraru, Katarzyna Aleksandra Jalowiec, Cesare Medri, Michael Daskalakis, Sacha Sergio Zeerleder, Behrouz Mansouri Taleghani

**Affiliations:** 1 Department of Hematology and Central Hematology Laboratory, Inselspital, Bern University Hospital, University of Bern, 3010 Bern, Switzerland; annina.capraru@insel.ch (A.C.); katarzynaaleksandra.jalowiec@insel.ch (K.A.J.); cesare.medri@insel.ch (C.M.); michael.daskalakis@insel.ch (M.D.); sacha.zeerleder@insel.ch (S.S.Z.); 2 Department of Biomedical Research, University of Bern, 3010 Bern, Switzerland; 3 Department of Immunopathology, Sanquin Research and Landsteiner Laboratory, 1006 AD Amsterdam, The Netherlands

**Keywords:** platelet transfusion guidelines, platelet transfusion alternatives, platelet additive solutions, platelet pathogen inactivation/reduction, cold stored platelet concentrates

## Abstract

Since the late sixties, therapeutic or prophylactic platelet transfusion has been used to relieve hemorrhagic complications of patients with, e.g., thrombocytopenia, platelet dysfunction, and injuries, and is an essential part of the supportive care in high dose chemotherapy. Current and upcoming advances will significantly affect present standards. We focus on specific issues, including the comparison of buffy-coat (BPC) and apheresis platelet concentrates (APC); plasma additive solutions (PAS); further measures for improvement of platelet storage quality; pathogen inactivation; and cold storage of platelets. The objective of this article is to give insights from current practice to future development on platelet transfusion, focusing on these selected issues, which have a potentially major impact on forthcoming guidelines.

## 1. Introduction

In 1910, William Duke discovered the link between platelet count and bleeding time of patients as well as cessation of bleeding in association with low platelet numbers after transfusion of fresh whole blood [1]. However, it took four more decades until selective platelet transfusion (PT) became available. PT relieved hemorrhagic complications of thrombocytopenic leukemia patients and hence confirmed the key role of thrombocytopenia in this context [2,3].

In the following years, PT was increasingly implemented in routine blood transfusion strategies in different clinical settings, either to stop (therapeutic strategy) or prevent (prophylactic strategy) actual bleeding. PT is consensually accepted for severely thrombocytopenic patients receiving chemotherapy or undergoing surgery or invasive exploration. However, all situations require different transfusion protocols, and individual decisions depend on the type of pathology and the patient’s characteristics. Various national specific societies and/or competent authorities have published guidelines for platelet transfusion [4,5], but international and consensual guidelines are lacking. Therefore, transfusion protocols often vary from country to country, and—in spite of given national guidelines—still often also vary from hospital to hospital or even from team to team.

International variation of practice guidelines may in part be due to differences of platelet concentrates in use. For instance, according to given standards in different countries and regions of the world, single units of platelet concentrate (PC) may contain from 2 to 8 × 10^11^ platelets. Moreover, there are further differences, e.g., in preparation techniques, additive solutions in use, maximum storage duration, and implementation of pathogen reduction/inactivation processes. Therefore, it is often difficult to compare published results and recommendations, as the quantity and quality of platelets in a bag and the definition of a PC “unit” is not always mentioned and may vary extensively. Finally, as in all fields of therapy, current and upcoming advances will significantly affect present standards.

The objective of this article is to give insight into and discuss selected issues being currently debated and/or that have or may have a major impact on future guidelines for PT. At the end of each of the five sections (apheresis versus pool platelets/platelet storage media/platelet pathogen inactivation/cool storage and frozen platelets/alternatives to platelet transfusion), the reader will find a specific summary.

## 2. Methods

We searched for specific publications in PubMed and MEDLINE. We applied no restrictions to language and publication date. We performed the last search on 30 September 2020. Per topic, at least two authors screened the obtained references and selected those to be considered in this manuscript. Results per topic and their discussion are included in the following specific sections.

## 3. Results

### 3.1. Apheresis Versus Pool Platelets

There are three methods of platelet collection for transfusions. Two of them are based on the donation of whole blood (WB), which is subsequently centrifuged, resulting in separated blood components according to density. An initial soft-spin centrifugation step results in a product containing RBCs in the lower part and the platelet rich plasma (PRP) in the upper part of the collection bag. Using a hard-spin centrifugation step initially, three layers are obtained: the RBCs, the buffy coat (BC) (mainly leukocytes and platelets), and the plasma. After pooling the PRP or BC from 4–6 donors of the same AB0 blood group, a second hard-spin centrifugation of the PRP separates platelets from plasma (PRP-PC), whereas in the pooled BC a second soft-spin centrifugation separates the majority of leukocytes and RBCs from platelets. Finally, the gained BC-platelets are re-suspended in donors’ plasma or in a mixture of additive solution and plasma (BC-PC). The BC-PC are the preferred pool PC in Europe and Canada, whereas PRP-PC is favoured in the U.S. and a few other countries [6,7]. The third method for platelet collection is the production of single-donor platelet concentrates by apheresis (A-PC). During this centrifugation-based cell separation process, the platelet-layer (including some amount of plasma) is collected, while the remaining blood constituents are returned to the donor. Storage of PLT in plasma is common in countries without or with low usage of pathogen reduction technology, while usage of additive solution is practiced in countries using pathogen-reduced APC or PPC. In Europe, all PC are leukocyte-depleted prior to storage in order to reduce side effects [8]. PRP-PC are leukocyte-depleted before or after storage. The usual shelf life of PC is 5 days and may be extended to 7 days in case of pathogen-inactivated products. There are countries like the U.S. or Germany, where pathogen inactivation is not yet routinely applied. The distribution of the different PCs varies in different countries. Kuwait and the Japanese Red Cross are using exclusively apheresis PCs; in the United States the distribution of apheresis platelets comprised 91.3% of all platelets in 2017 [9]. In Europe the distribution of whole blood PCs and apheresis PCs varies between different countries; e.g., the U.K. produces about 90% apheresis platelets, whereas countries like Finland, Denmark, The Netherlands, Croatia, Cyprus, and Israel have focused mainly on the production of whole-blood derived PCs [10].

According to, e.g., current American guidelines, available comparative studies consider these types of PCs to be clinically equivalent [4]. Thus, in routine circumstances, they can be used interchangeably. One acknowledged exception is single-donor platelets from selected donors, which are necessary for patients requiring HLA (human leukocyte antigen) compatible and/or HPA (human platelet antigen) compatible platelets, i.e., mainly those with platelet refractoriness due to HLA and/or HPA antibodies as well as in neonatal allo-immune thrombocytopenia (NAIT)). Nevertheless, some centres still prefer apheresis PCs for patients with haematological diseases, e.g., in order to reduce donor exposure. In many countries, pooled PCs are less costly, which may also have influence in opposite decision-making. In the following sections, we summarize current evidence for using pool or single-donor PCs.

There are various differences between apheresis and pool platelets and their potential significance, including the following:

- Several studies have shown that transfusion of apheresis platelets results in higher corrected count increments (CCIs) in vivo compared to BC- or PRP-PCs [11,12]. However, a higher CCI may not result in an improved haemostatic effect or bleeding prevention, as shown also in a different context by Triulzi et al. [13].
- The risk of bacterial contamination of PC may be lowest in A-PC, since the procedure implies only one donor and one venipuncture, in contrast to pooled PC, where four to six donors and venipunctures are necessary. However, the estimated contamination rates of the different platelet products partly overlapped and partly varied widely in the different studies, depending on the time of sampling and the bacterial detection method in use [12,14,15,16,17].
- Regarding the risk of transmission of viral infections, older studies reported at least a twofold increased risk for HIV (human immune deficiency virus), HCV (hepatitis C virus), and HBV (hepatitis B virus) associated with pooled PCs [17], whereas more recent data from the German National Blood Donor Surveillance System showed an overall comparable estimated residual risk of transmission for pooled and single-donor PCs for the years 2006–2012 [18]. The implementation of pathogen inactivation processes will equalize potential differences and sustainably reduce the rates in microbial transmission for the different PC products globally in the next decades (see separate section).
- Rhesus-D alloimmunization resulting from PC transfusion is of particular importance in female children and female adults of childbearing potential being treated with curative intent. The volume of contaminating RBCs is lower for A-PC compared to pool PCs. However, several studies have shown that neither cumulative PLT dose nor repeated exposure to Rhesus-D-positive platelet concentrates is associated with a higher rate of anti-D alloimmunization in none of the platelet products [19,20,21,22,23]. Prevention of RhD alloimmunization resulting from platelet transfusions to RhD-negative recipients can be achieved either through the exclusive use of platelet products collected from RhD-negative donors or via anti-D immunoprophylaxis [4].
- Platelets express HLA class I and HPA antigens. In the majority of cases, platelet refractoriness is caused by alloimmunization against HLA antigens; it is less frequently due to HPA-alloantibodies [24]. However, results of the “Trial to Reduce Alloimmunization to Platelets Study Group” did not show differences between leukoreduced pool PC and A-PC groups according the development of HLA antibodies or allo-immune platelet refractoriness [25]. The Study group could demonstrate that leukoreduction is the major factor associated with reduction of HLA alloimmunization after platelet transfusion and not the product type of platelets or the number of donors that the patient was exposed to.
- With regard to adverse events after platelet transfusion, several studies investigated the incidence of transfusion-related acute lung injury (TRALI) after platelet transfusion [26]. There was no evidence of an increased risk of TRALI caused by pool PCs compared to A-PCs in either of the reports [27,28]. Febrile non-hemolytic transfusion reactions (FNHTRs) are mainly due to the accumulation of leukocyte- and platelet-derived cytokines during platelet storage and also due to in vivo cytokine production caused by recipient antibodies to transfused donor’s leukocytes. Several studies demonstrate that the rate of FNHTRs is equal for both platelet products (pool PCs and A-PCs) when the products were leukoreduced [11].
- To investigate how PC characteristics (such as dose, source, and storage duration) influence the rate of transfusion-related adverse events (including allergic/hypersensitivity reaction, sinus bradycardia or tachycardia, hypertension, hypotension, dyspnoea, hypoxia, wheezing, cough, haemolysis, rigor/chills, fever, and infection), Kaufman et al. performed a sub-analysis of the before-mentioned study by Triulzi et al. The type of platelet product (pool PC versus A-PC) did not significantly influence the risk of adverse events; only the platelet dose was associated with the rate of adverse reactions [29].

In summary, BC-PC, PRP-PC, and A-PC reveal similar quality, haemostatic benefits, and adverse event profiles. Hence, they are considered clinically equivalent for most the patients. However, A-PC from selected donors remains essential when HLA/HPA compatible platelet transfusions are needed.

### 3.2. Platelet Storage Media

Historically, platelets were stored in plasma, in order to preserve their hemostatic and structural integrity during storage [30]. However, transfusion of plasma is always associated with a significant risk of allergic reactions, circulatory overload, and passive transfer of anti-A and anti-B-antibodies. In the 1980s, plasma was suspected to contain harmful enzymes that caused the so-called platelet storage lesion, which triggered the development of different types of platelet additive solutions (PASs) to replace plasma as the sole storage medium and reduce costs [31]. Currently used storage media typically contain 50–80% PAS, with a corresponding plasma-remainder of 20–50% [32]. Recent approaches successfully reduced the latter to 5% [33]. Transfusion of PC in PAS reduced the frequency of allergic transfusion reactions by nearly 50%. Because it also preserves adequate platelet increments, PAS has increasingly become the preferred storage medium [34]. Research is ongoing to define the optimal composition of platelet storage media in order to modulate the storage quality, facilitating the longest possible shelf life together with a minimal loss of functionality and effectiveness not inferior to plasma-only stored platelets.

#### 3.2.1. Components of PAS

Platelet additive solutions aim to preserve platelet function over time and to prevent storage lesions [32]. All types of PAS contain NaCl to preserve osmolality, citrate to prevent calcium-induced clumping, and in almost all types, acetate to replace glucose as an additional source of energy for platelets. Acetate reduces synthesis of lactic acid and serves as a catcher of H+ ions from the environment, thus preventing the detrimental lowering of pH. Moreover, oxidation of acetate produces bicarbonate, which serves as an efficient buffer, further preventing a decrease in pH. Although glucose remains crucial for the metabolism of platelets, it is not added in all types of PAS, since the amount of glucose in the remainder plasma-fraction of currently used PASs (usually 20–50%) seems to be sufficient. Moreover, the addition of glucose increasingly provokes its caramelisation during steam sterilization that occurs as part of the manufacturing process of PASs. However, in the case of less than 15–20% plasma-fraction, the quantity of glucose becomes progressively insufficient for sustained preservation of the platelets’ metabolism. The adding of phosphate also seems to prevent the decrease of pH (buffer) and to stimulate platelet glycolysis to increase production of lactic acid. Additionally to its effect on coagulation through the chelation of divalent cations such as calcium and magnesium, citrate may modify potassium efflux through the platelet membrane. The addition of electrolytes like potassium and magnesium to some types of PAS results in improved pH preservation and lowering of platelet activation markers [30].

#### 3.2.2. Types of PAS

Present generations of PASs are categorized from A to G (PAS-A to PAS-G) based on their composition [32]. As an alternative, other names can be used, for instance InterSol for PAS-C, SSP+ for PAS-E, and Plasma Lyte A for PAS-F. This nomenclature was developed to define concentrations of citrate, phosphate, acetate, magnesium, potassium, gluconate, and glucose. Describing all types of PAS is beyond the scope of this study and is summarized in the referenced reviews. Here, we will focus on selected types of PAS and their most important properties. For PAS-B (containing only citrate and acetate) and PAS-C (containing citrate, acetate, and phosphate), corrected platelet count increments are lower as compared to PAS-E (containing citrate, phosphate, acetate, magnesium, and potassium) but comparable to plasma-only stored platelets. So far, platelet increment data is best for PAS-E. Studies using the recently developed PAS-F (containing acetate, magnesium, and potassium as key constituents) showed that platelets might be stored for 13 days with recovery and survival outcomes being equal or even superior to 7-day-stored platelets in plasma [35].

To summarise, an ideal storage medium does not exist to-date. There are myriad advantages and disadvantages of plasma vs. PAS stored platelets. Nevertheless, current PAS generations may extend shelf life up to 13 days, retaining adequate platelet increment in the case of PAS-E and recovery and survival data in the case of PAS-F. In addition, there is a decrease in adverse reactions related to plasma. Further development of PAS types should aim to assure even better platelet quality and to facilitate pathogen inactivation as well as cold storage of platelets. Because of preserved quality and efficiency, along with reduced transfusion-related adverse events as well as costs of PC manufacturing, PASs are steadily replacing plasma as the storage medium of platelets.

### 3.3. Pathogen Inactivation of Platelet Concentrates

Safety measures regarding the risk of pathogen transmission are implemented throughout the complete manufacturing process of blood components for transfusion [36]. This led to an overall impressive improvement in infectious risks of blood transfusion in the last two decades. However, in many countries bacterial contamination of platelet concentrates (PCs) remained as the most prevalent transfusion-associated infectious risk, being responsible for considerable morbidity and mortality in blood transfusion. The UN National Safety Network rated the frequency of septic transfusion reactions to one per 41,000 to 116,000 distributed PC between the years 2010 and 2016, with Staphylococcus and Streptococcus infections being the most often-detected pathogens [37].

The main reason for this is that, thus far, platelets are mainly stored at room temperature (22 ± 2 °C) (RT) to maintain their optimal viability and function. However, this provides a favorable environment for bacterial growth, with an increasing risk during their storage. Therefore, the shelf life of RT-PC is currently limited to 3–7 days, depending on the national specifications, usually stipulated by responsible authorities. The “cold storage” of platelet products is described in a separate section of this article; it may prolong storage time up to 21 days (4 °C) or even two years (−80 °C).

Bacterial detection methods are useful but not sufficiently sensitive because of the initial low bacterial concentration [38,39]. Furthermore, undetected bacteria may grow during storage time into health-threatening levels [40]. Therefore, the development of pathogen reduction technology systems are a major step in transfusion medicine by reducing the risk of transfusion-transmitted diseases. Currently, three commercial systems (Intercept^®^, Cerus, Concord, CA, USA; Mirasol^®^, Terumo BCT, Lakewood, CO, USA; and Theraflex^®^, Macopharma, Tourcoing, France) are approved by several national competent authorities and available for routine use. Recent in vitro experiments showed pathogen inactivation to be applicable in cold-stored PC (see separate section) and will probably be soon included in larger clinical trials [41].

Intercept Blood System (Cerus) (IBS) was the first approved pathogen inactivating system in clinical use. As described elsewhere, the procedure is based on the addition of the psoralen compound amotosalen HCl to the PC suspended in 65% PAS and 35% plasma, resulting in an amotosalen concentration of 150 μmol/L [42]. The amotosalen intercalates between the pyrimidine base pairs of nucleic acids. Upon UVA illumination with an energy of 3 J/cm^2^ at 320–400 nm wavelength, it forms covalent bonds, preventing further replication and thus inactivating pathogens as well as residual leukocytes. A compound absorption device reduces the amotosalen residual content to <2 μmol/L in the final PC product for transfusion, which can be stored for up to 7 days [42,43]. National hemovigilance programs from Switzerland, France, and Belgium reported a significantly lower rate of septic reactions when using IBS pathogen-inactivated platelets between 2005 and 2016 [36].

One of the main and early concerns and topic of many studies was the assumed higher occurrence of bleeding events in patients transfused with IBS, or more generally, pathogen-inactivated platelets [44,45,46]. However, the most recent analyses found no significant difference between patients receiving pathogen-reduced (IBS and Mirasol) platelets and the control group, including the occurrence of clinically significant/severe bleedings, all-cause mortality, and even severe transfusion reactions [47,48]. Comparing IBS platelets with platelets in PAS and platelets in plasma showed that IBS platelets were non-inferior to platelets in PAS regarding bleeding events WHO Grade 2 or higher but were inferior to platelets in plasma [49]. Another meta-analysis investigated the efficacy of IBS platelets in patients with chronic cytopenia due to bone marrow failure, since this patient collective is transfusion-dependent and often immunosuppressed. The data described a lower increment in the thrombocyte count compared to untreated platelets but without a higher bleeding rate [50]. All data indicated that IBS treated platelets result in a lower platelet increment. Butler et al. additionally reported a 7% increase of IBS-PC transfusions and a shorter time interval between transfusions in their systematic review [51]. The already introduced meta-analysis of Estcourt et al. could reproduce all the results [47].

Trying to explain the reduced platelet increment and slightly higher bleeding rate of WHO Grade 2 or less, some groups studied IBS treated platelets extensively in vitro, including adhesion to collagen and vWF under flow and different proteomics analysis, showing impairment of platelets physiologic activity through IBS exposition. Treated platelets seem to have a reduced response to certain agonists (vWF, collagen, thrombin) due to loss of surface proteins, needed for interaction. IBS treated platelet transfusion in combined immune-deficient mice revealed a faster platelet degradation. IBS treated platelets have upregulated apoptosis pathways, which can explain their accelerated degradation [52].

Altogether, IBS reliably reduces infectious risks from platelet transfusion while maintaining adequate efficacy. Switzerland recently confirmed the above-mentioned study results by analyzing the data of their mandatory national hemovigilance system [42]. In 2011, it was the worldwide first country switching mandatorily from untreated platelets towards 100% IBS-PC in PAS. Between 2005 and 2011, there were 16 documented transfusion-transmitted bacterial infections, including three fatalities. Since 2011, there have been no reported bacterial-transmitted transfusion reactions; additionally, there has been a specific and overall statistically significant reduction of transfusion reactions. Even though there was an expected loss of 10–15% of average platelet content per unit, there was no increase in IBS-PC requirements, no increased bleeding complication, and no ineffectiveness of transfused IBS-PC reported [42].

In vitro comparison of cryopreserved IBS-PC and control PC showed that IBS-PC appeared slightly more susceptible to lesion effects by freezing than conventional PC, in particular in assays on day one after thawing. However, these differences were small in relation to the dramatic effects of the freezing process itself. Moreover, functional tests, including coagulometry and rotational thromboelastometry, showed similar results [53].

Mirasol Pathogen reduction technology system (TerumoBCT) (MPR) was FDA approved in 2007. It uses riboflavin (vitamin B2), which has a very high safety profile and a narrow spectrum UV light (280–360 nm), to inhibit proliferation of bacteria, viruses, and white blood cells [54,55]. The principle of action is similar to Psoralen, as Riboflavin intercalates with the DNA/RNA bases and becomes chemically active through UV light. Electron transfer and the production of singlet oxygen and hydrogen peroxide are the results of the provoked photochemical reactions, subsequently causing base damage and strand breaks [56]. There are several UV light spectra described as being successful pathogen inactivators. When analyzing the effectiveness and side-effect profile of narrow and broad UV light, it seems that narrow light is more suitable for bacterial and viral inactivation, while preserving platelet qualities and metabolism even though a significant reduction of platelet counts was observed. Light energy should also be chosen wisely, since increased light energy effectively inactivates pathogens, while causing a higher degradation of platelets. Thus, the latter is not recommended [57,58].

One of the main questions regarding MPR technology is its degree of negative impact on the physiologic hemostatic function of platelets, which may result in increased frequency of bleedings of WHO Grade 2 or higher. A recent non-inferiority multicenter controlled trial compared the transfusion of MPR-PC and standard PC in plasma. The primary outcome parameter was the proportion of transfusion treatment periods in which the patient had Grade 2 or higher bleeding, as defined by WHO criteria. There was a 3% absolute difference in Grade 2 or higher bleeding in the intention-to-treat analysis: 51% of the transfusion treatment periods in the control arm and 54% in the intervention arm, with significance for non-inferiority (*p* = 0.012). However, in the per-protocol analysis, the difference in Grade 2 or higher bleeding was 8%: 44% in the control arm and 52% in the intervention arm, with a failed significance of *p* = 0.19 for non-inferiority [59]. Therefore, the study indicated a slightly higher incidence of bleedings of WHO Grade 2 or higher in the MPR-PC group. MPR-PC had a lower platelet increment than the untreated control group. The transfusion increment was about 50% lower in the MPR thrombocyte treated group compared to the control group [58]. Similar biochemical findings are reported for MPR- and IBS-PC. MPR seems to activate apoptosis pathways, responsible for earlier destruction of platelets [60].

Alloimmunization is one of the main problems for multiple transfused patients, causing a decreased increment after transfusion and thus higher bleeding risk. The question thus arises whether pathogen reduced platelets are prone to trigger alloimmunization as part of the pathomechanisms of lowered platelet increment. A multivariate study analyzed this question by measuring anti HLA Class I and II antibodies before and after transfusion of MPR platelets and untreated control platelets. All patients lacked alloantibodies at randomization. HLA Class I antibodies were detected more often after transfusion of MPR platelets, while HLA Class II antibodies were detected in both groups with similar frequencies. HLA Class I antibodies are probably caused by a platelet-mediated indirect immunization pathway. The differences between groups regarding high-titer Class I antibodies are less pronounced, so that the clinical implications of these findings are reduced and need further analysis. MPR-PC surely do not protect alloimmunization [61]. Another study of HLA Class I alloimmunization after transfusion of MRP-PC and control PC in plasma showed no significant difference between groups [58].

MPR has also been used successfully in cryopreserved platelets. In vitro testing showed that the platelets had a better hemostatic activity and fewer morphological changes than the untreated control group and maintained their function. On the other hand, they seemed to have a higher metabolic activity; further studies are under way [62].

Theraflex UV-Platelet System (MacoPharma) is the most recently introduced pathogen inactivation technology. It differs from the former two by using ultraviolet C light (UVC) only, i.e., without the need for a photoactive substance to interfere with the nucleic acids. UVC interacts directly with nucleic acids, resulting in the dimerization of pyrimidine bases, thus preventing further replication of the DNA/RNA. Theraflex inactivates bacteria, viruses, and protozoa replication as well as contaminating white blood cells. Available studies showed a broad effectiveness, with a bacterial load reduction of 3–7 log [63]. It also effectively inactivates the majority of coated and non-coated viruses (including HIV) with ≥2 log [64].

Theraflex was very recently evaluated in a phase III clinical trial (CAPTURE), and the results were published online in February 2021 [65]. In a randomized, controlled, double blind, multicenter, non-inferiority trial, the group compared the efficacy and safety of UVC-treated platelets (UVC-PC) to that of untreated platelets in thrombocytopenic patients with hematologic-oncologic diseases. The primary objective was to determine the non-inferiority of UVC-PC, assessed by the 1 h corrected count increment (CCI) in up to eight per-protocol platelet transfusion episodes. The defined non-inferiority margin of 30% of UVC-PC was narrowly missed, as the mean differences in 1 h CCI between standard platelets versus UVC-PC for intention-to-treat and per-protocol analyses were 18.2% (95% confidence interval [CI]: 6.4%; 30.1%) and 18.7% (95% CI: 6.3%; 31.1%), respectively. Moreover, the UVC group had a 19.2% lower mean 24 h CCI and received about 25% more PC units, but the average number of days to next platelet transfusion did not differ significantly between treatment groups. The frequency of low-grade adverse events was slightly higher in the UVC-PC group, and the frequencies of refractoriness to platelet transfusion, platelet alloimmunization, severe bleeding events, and red blood cell transfusions were comparable between groups. The study suggests that transfusion of pathogen-reduced platelets produced with the UVC technology is safe; however, non-inferiority was not demonstrated.

Hepatitis E virus is one of the non-enveloped viruses with increasing incidence in industrialized countries and may have serious clinical courses with chronic hepatitis and neurological complications, particularly in immunocompromised patients [66]. Mini pool nucleic acid amplification testing is currently used in blood manufacturing establishments for screening, but it can only detect high viremia [67]. IBS was reported to be ineffective to neutralize hepatitis E virus [68,69]. MPR has been proven effective, showing a reduction of viral load of 2–3 log [70]. The UVC method was highly effective, since the standard dose decreased the viral load below the standard of detection [63].

Initial in vitro studies have already showed the effectiveness of this method in cryopreserved platelets. Blood group AB0 matched pool PC were treated with Theraflex before freezing. In vitro analysis after thawing showed that pathogen-inactivated platelets and untreated controls expressed similar amounts of adhesion molecules, but treated platelets expressed a higher level of activated GPIIb/IIIa, which may indicate a higher susceptibility to damage during cryopreservation. Nevertheless, the treated platelets showed no difference to the controls regarding aggregometry, thromboelastography, and thrombin generation assays [71].

To summarise, there are three pathogen reduction technologies for PC, with two of them (IBS and MPR) being available in some countries for more than a decade for routine use. This approach is increasingly becoming the new paradigm in transfusion safety, as the described technologies are capable of diminishing or neutralizing infectious threats, including those that are not addressed or may not be detected by standard screening techniques. In addition, gamma or X-ray irradiation of platelet units for GvHD-prophylaxis becomes superfluous, because all described technologies already inactivate white blood cells efficiently.

### 3.4. Cold and Frozen Storage of Platelet Products

Platelet concentrates are currently mainly stored in gas-permeable bags with increased surface at 22 ± 2 °C (room temperature, RT) and constant agitation. Depending on the country, the accepted shelf life is limited to 3–7 days, mainly due to bacterial proliferation risk and progressive metabolic processes. However, even in the case of accepting a shelf life of 7 days, supply and logistic challenges and problems remain significant. To address and overcome the obvious limitations, alternative storage methods, including lyophilized (freeze-drying), cryopreserved (freezing with DMSO at −80 °C), and cold-stored platelets (CSPs), are currently being studied in clinical trials.

Until 1980, platelets (PLTs) were stored at 4 °C (“cold stored platelets”). However, this method was abandoned in light of a study conducted by Murphy et al., demonstrating that the half-life of cold-stored platelets after transfusion was markedly reduced in blood circulation (1.3 days compared to 3.9 days of RT stored PLTs) and that they undergo morphological distortions [72]. The latter are described as cold storage lesions and comprise irreversible disc-to-sphere transformation, apoptosis, signs of activation with increased expression of both P-selectin and GPIba, and increased production of thromboxane A2 [73]. This led to a paradigm shift towards storage of PC at RT. However, because the cold storage lesions include PLT pre-activation, cold stored PLTs may have a better hemostatic effectiveness for treating active hemorrhage than RT stored PLTs. Becker et al. demonstrated that CSPs were not inferior to RT PLTs, showing even greater reduction of bleeding time in healthy volunteers treated with aspirin [74]. In 2017, the US Food and Drug Administration (FDA) stated that apheresis PLTs stored at 4 °C for up to 72 h could be used for the treatment of active hemorrhage.

Two recent studies addressed the characteristics of CSPs in more detail. Nair et al. demonstrated that CSPs form a significantly stiffer and stronger blood clot with more crosslinks as well as thinner, denser, and straighter fibers compared to RT stored PLTs [75]. Additionally, cold-induced binding of plasma factor XIII to the platelet surface resulted in enhanced mechanical clot strength by increased crosslinking [74]. The study of Johnson et al. focused on the biochemical and morphological changes platelets undergo during cold storage [76]. CSPs had a lower metabolic activity, using ATP mainly for shape changes from disc to sphere. According to the maintained metabolic reserves and shape-change capacity, CSPs were at least non-inferior in comparison with RT-PC [75]. Both studies agree that CSPs are a useful alternative to RT platelets and could be even more effective in a post-traumatic context, where speed and strength of clot formation has priority. A recent pilot trial supports the feasibility of platelets stored cold for up to 14 days and provides critical guidance for future pivotal trials in high-risk cardiothoracic bleeding patients [77].

Cryopreserved Platelets (CPPs) carry minimal bacterial contamination risk and can be stored at −80 °C for several years. Stored as platelet pellets at −15 °C, the first application of CPPs was in pediatric patients with thrombocytopenia and resulted in a moderate success [78]. After invention of the dimethyl sulfoxide (DMSO) method by Valeri and colleagues in 1974 [79] and its modifications [80,81,82], CPPs can be stored at −80 °C, resulting in a more standardized and frequent manufacturing of CPPs, particularly used by military services. Further investigations showed that freezing and thawing affects the morphology and surface marker expression of CPPs, resulting in a pre-activation state [75]. In several trials in bleeding patients, CPPs revealed equivalent or even slightly improved hemostatic effectiveness when compared to RT-stored PLTs [41,83,84,85]. Serious adverse events, including an increased risk of thrombo-embolism, could not be found. However, there is an increased release of biological response modifiers in CPPs, which may be a potential further clinical risk of these products [86]. Combining IBS pathogen inactivation and cryopreservation did not affect the release of immunomodulatory factors more than cryopreservation alone [87]. CPPs showed lower posttransfusion increments and reduced 24 h recovery, making them more applicable for resuscitation of active hemorrhage and less suitable for prophylactic transfusion in thrombocytopenic patients.

In highly alloimmunized thrombocytopenic patients after chemotherapy, autologous CPPs can serve as an additional source of HLA-compatible units. A Swiss group used autologous CPPs for thrombocytopenic alloimmunized and PLT-refractory patients undergoing chemotherapy. They reported a significantly higher median 1 h PLT count increment compared to RT-stored, AB0-matched, but HLA-unselected PCs [88].

The largest and most recent randomized, double blind, multicenter pilot study of CPPs to date, the CLIP-I trial, investigated the effects of CPPs versus RT stored PLTs in 121 cardiac surgery patients [89]. The trial’s primary endpoint of feasibility was met, and investigators found neither significant differences between the two patient cohorts in blood loss or RBC use nor in adverse event rates [86].

In summary, CPPs could be a useful addition for certain patient settings and populations. Frequent usage in frontline military surgical units and in rural hospitals as well as “back-up units” in busy urban trauma centers are reasonable scenarios. For HLA-alloimmunized patients, autologous and allogeneic CPPs could expand the number and variety of appropriate PCs.

The production of lyophilized (synonymous: freeze-dried) platelets (LPs) was already attempted in the 1950s. However, the rehydrated PLT units showed no hemostatic effectiveness [90]. In 1995, Read and colleagues reported an improvement of the lyophilization procedure by stabilizing the PLTs with paraformaldehyde (pLPs) before freeze-drying [91]. In vitro testing of rehydrated pLPs revealed normal morphology, with adhesive properties remaining largely intact. Even though activation and aggregation functions are impaired, they retain the ability to facilitate coagulation [92,93,94]. In animal studies, the infusion of pLPs improved bleeding time and showed no difference with respect to transfusion requirements, PLT increment, adverse effects, or survival in comparison to fresh PLTs [88,95]. There is no evidence supporting an increased risk of pro-thrombotic complications after transfusion of pLPs. In vivo studies showed that pLPs have minimal adhesion to intact endothelium and are rapidly cleared from blood circulation if they are not bound immediately to the site of injury after transfusion [96,97].

In 2001, Wolkers and colleagues described another procedure, where PLTs were loaded with trehalose before lyophilization [98]. After rehydration, these PLTs showed a recovery rate of ca. 80%, and about 40% of the cells showed characteristics of pre-activation [99]. Compared to fresh PLTs, their clot formation time was similar, with an aggregation rate ca. 80% and aggregation speed ca. 40%.

The current LPs have inferior hemostatic properties compared to fresh PLTs, but they may be an alternative if fresh PLTs are not available, especially in the setting of acute hemorrhage. Due to their rapid clearance from blood circulation, they are not an alternative for prophylactic transfusions in thrombocytopenic patients.

In summary, cold and frozen stored platelet products could be an efficient substitute or at least supplementation of RT-stored PCs. In vitro results demonstrate that their clotting and hemostatic properties could exceed those of conventional PCs, though sufficient evidence based on clinical studies in routine use is still pending. It has reasonably been postulated that the “one size fits all” strategy, where only RT-stored PCs are used for both prophylaxis and therapeutic needs, may be neither cost effective nor the best choice for the single patient. The task of switching from “RT PC only” supply to a dual inventory with CSP and RT PC is obviously huge. However, recent pilot studies have shown the feasibility of building and maintaining a dual inventory and the efficacy of CSPs in patients undergoing complex cardiothoracic surgery [76]. The challenge of a dual inventory may be mitigated by storing platelets at room temperature until they are close to the expiration date and only then refrigerating them [100].

The data for cryopreserved and lyophilized platelets are currently not sufficient to suggest a paradigm shift but are extremely promising and interesting, suggesting that further studies should be done to assess the efficacy and feasibility of these fascinating blood products.

### 3.5. Alternatives to Platelet Transfusions

The increasing demand for platelet products, in combination with their limited storage time, outpaces the availability of donors, resulting in a high economic burden and product shortage. In addition to the potential transmission of viruses and bacteria, platelet transfusion is associated with potentially life-threatening complications, such as allergic and febrile reactions. In addition, alloimmunization after repetitive platelet transfusion may result in platelet refractoriness and hence with increased risk for hemorrhagic complications for affected patients due to a significantly constricted number of compatible products. Recent attempts to produce platelets devoid of HLA Class I molecules are promising. Such an approach would offer a solution to prevent alloimmunization and reduce the risk of refractoriness [101]. However, this will not provide a solution to meet the increasing needs of platelet products and hence platelet donors. Therefore, it may be prudent to find substitutes for platelets products.

Alternatives to platelet transfusion in clinical use include auxiliary therapeutic strategies to support plasma coagulation. Such strategies are of special value in the treatment of bleeding complications of thrombocytopenic patients, since clots with low platelet contents are more prone to lysis. Platelets contain plasminogen activator inhibitor-1 (PAI-1) and Factor XIII, which inhibit fibrin degradation and stabilize the clot firmness through fibrin cross-linking, respectively [102].

Thrombopoietin (TPO) mimetics are used off label in a wide range of malignant diseases with impaired thrombocyte production due to the disease itself and/or the therapy applied. Their extended indication may be an appropriate approach to avoid or at least reduce platelet transfusions. For example, there is some evidence for the usage of TPO agonists in myelodysplastic syndromes. A meta-analysis including 384 patients described a lower bleeding and transfusion rate in TPO treated patients compared to placebo [103]. However, this treatment is expensive, and some authors assume an increased risk of thrombosis upon TPO agonist treatment [104]. TPO agonists are most suitable to increase platelet count in the long term (i.e., days to weeks). Hence, they are not appropriate for the treatment of acute bleedings in thrombocytopenic patients.

Research activities focusing on alternatives to platelet transfusion have shifted into the foreground in the last decades. They attempt to find an effective “off the shelf” product, with optimal storage properties, that can mimic the biochemical platelet mechanisms (adhesion, aggregation, and coagulation) even after prolonged storage, but with no need for blood group typing and a reduced risk of side effects. In 1992, an autologous, semiartificial alternative to platelet transfusion, called “Thromboerythrocytes”, was reported. Peptide sequences containing the specific recognition sequence, containing arginine-glycine-aspartic acid (RGD) as found in the extracellular matrix and in fibrinogen, were cross-linked to amino groups on erythrocytes. The coupled RGD sequences revealed interaction with the GPIIb/IIIa receptor in fluid phase as well as binding to activated platelets adherent to collagen. The authors reported of their in vitro studies, indicating that 50 mL of processed thromboerythrocytes may be equivalent to at least two PC units [105]. Although a very attractive concept, thromboerythrocytes have not found their way into the clinic thus far. A comparable approach by means of membrane modification, called “Plateletsomes”, was successfully used in rat experiments. In this case, platelet membrane fractions, including relevant platelet-receptors such as GPIb, GPIIb-IIIa, and GPIV/III, were extracted from platelets and incorporated into liposome membranes. These plateletsomes reduced tail bleeding in thrombocytopenic rats by 67% [106].

Given the fact that even small membrane fragments seem to have comparable hemostatic qualities to intact platelets, Cypress Bioscience Incorporated (San Diego, CA, USA) developed concentrates of platelet fragments of non-viable platelets, called Infusible Platelet Membranes (IPMs). First, fresh, or even expired platelet concentrates are centrifuged and washed to remove plasma and contaminating white and red blood cells. Subsequently, they are pasteurized to reduce bacterial contamination. Finally, their sonication yields spherical vesicles. When stored at 4 °C, the IPM product is stable over two years. In healthy volunteers, IPM did not alter coagulation parameters, was tolerated well, and importantly did not show any immunogenicity. Indeed, administration of IPM resulted in an improvement or cessation of bleeding in thrombocytopenic patients with moderate bleeding. Interestingly, a patient refractory to platelet transfusion, who did not respond to IPM, showed a platelet increment after consecutive platelet transfusions. There has been no phase III clinical trial for this product and no FDA approval due to difficulties in demonstrating its efficacy [107,108]. The lack of provable efficacy in patients has been attributed to its lack of GPIIb/IIIa epitopes in IPM preparations.

An attempt to improve the IPM idea resulted in “Thrombosomes”, entire membranes extracted from outdated platelets. However, in contrast to IPM, thrombosomes express platelet surface receptors, including GPIb, GPIIb/IIIa, and Annexin V. They efficiently adhere to collagen exposed in the subendothelial matrix, activate the tenase complex, and form a stable clot. In animal models for bleeding, thrombosomes reduced blood loss by 80% [109]. Thrombosomes are currently evaluated in clinical trials. One of the main technical challenges so far is the significant loss of relevant platelet glycoprotein receptors and hence functionality during thrombocyte membrane processing.

Another possible alternative to thrombocytes are liposomes, containing ADP and synthetic fibrinogen derived γ-chain inclosing a dodecapeptide motif. The liposomes accumulate at the intravascular lesion site and interact with platelets by binding of the fibrinogen γ-chain dodecapeptide sequence to GPIIb/IIIa and inducing the release of stored ADP, thereby further promoting platelet aggregation [110]. The efficacy of this synthetic platelet replacement has been demonstrated in thrombocytopenic rabbits: 60% of the animals were rescued after a potentially fatal liver hemorrhage. Treating thrombocytopenic rabbits before the induction of the liver hemorrhage showed a 100% survival rate. Importantly, no thromboembolic complications occurred [111,112]. Furthermore, in a bleeding model in rats, the liposomes accumulated at the venous lesion of the tails of thrombocytopenic rats, leading to reduced bleeding [107]. Pharmacokinetic studies in rats showed a predominant localization of the synthetic liposomes in the liver and spleen after 24 h, and after 7 days there was a complete clearance from all organs [113].

SynthoPlate are synthetic platelet nanoconstructs that integrate, in a heteromultivalent approach, the adhesion and aggregation process of platelets. SynthoPlate consists of a biocompatible liposomal membrane with, on one hand, von Willebrand Factor-binding peptide and collagen-binding peptide to mimic platelet adhesion, and on the other hand, GPIIb/IIIa to mimic platelet aggregation. The efficacy of SynthoPlate was demonstrated in a mouse model for liver injury and in a trauma model in pigs. SynthoPlate reduced blood loss, stabilized blood pressure, and improved survival [114,115].

A comparable approach finally resulted in the idea of completely synthetic “platelets” made from polymers incorporating both platelet properties—adhesion and aggregation. The nanospheres are composed of PLGA-PLL nanoparticles (poly(lactic-co-glycolicacid)-poly-L-lysine), conjugated to polyethylene glycol (PEG) and surface-bound fibrinogen-derived RGD peptides, with additional flanking amino acids to enhance adhesion. By binding through specific integrins like GPIIb/IIIa, activated platelets bind to RGD sequences [116]. In vitro assays show a high specificity of binding of the synthetic platelets to inactivated physiologic platelets, which might be an indicator for a low risk of thrombotic complications in vivo. In a traumatic bleeding model in rats, the administration of synthetic platelets before injury was proven approximately 25% more effective than recombinant Factor VIIa, which is currently used in uncontrolled hemorrhage [117,118]. The same kind of synthetic platelets improved survival in a mouse model with blast trauma [119].

HAPPI, an injectable hemostatic agent via polymer peptide interfusion, is one of the most recent developments. It is a polymer-based hemostatic agent that binds selectively to activated platelets and promotes their accumulation at the injury site. HAPPI consists of a hyaluronic acid backbone conjugated with a collagen-binding peptide and a von Willebrand factor-binding peptide. It is designed to bind to vWF and collagen on activated platelets, thus recruiting and activating additional platelets. Through lyophilization, HAPPI is stable at room temperature for several months. Administration of HAPPI in a thrombocytopenic mouse model with tail-vein laceration significantly reduced bleeding time and blood loss as compared to untreated mice. In rats with traumatic hemorrhage due to inferior vena cava rupture, HAPPI showed an almost threefold improvement in survival compared to those treated with saline [120].

A future-oriented strategy to reduce the shortness of platelet products is to propagate the in vitro production of thrombocytes through forward programming of human pluripotent stem cells to megakaryocytes using exogenously expressed transcription factors GATA1, LFI1, and TAL1. This approach is highly efficient, with a yield of up to 2 × 10^5^ megakaryocytes per activated human pluripotent stem cell. This technique could yield sufficient HLA-matched platelets to cover the needs for platelets [121]. The limitations of the technique are the high production costs and scarce availability of human embryonic stem cells [122,123].

In summary, current and upcoming advances will significantly affect present standards. In particular, B-PC and A-PC are considered clinically equivalent, revealing similar quality, effectiveness, and adverse event profiles. However, A-PC from selected donors remains essential when HLA/HPA compatibility of PC is needed. Current PAS generations may extend the shelf life of PC up 13 to days while retaining adequate platelet increment as well as platelet recovery and survival and decreasing adverse reactions. Because manufacturing costs are reduced simultaneously, PASs are steadily replacing plasma as the storage medium of platelets. There are three pathogen reduction technologies for PC, with two of them being available in some countries for routine use for many years. This approach is increasingly becoming the new paradigm in transfusion safety, because it safeguards against the majority of the remaining associated infectious threats and makes X-ray/gamma irradiation of PC (for prevention of GvHD) superfluous. Cold stored platelet products could be an efficient substitute or at least supplementation of PCs stored at 22 ± 2 °C. The in vitro hemostatic properties of these products may exceed those of the conventional PCs, at least in the therapeutic setting, i.e., the bleeding patients. Recent pilot studies have shown the feasibility of building and maintaining a dual inventory and the efficacy of the cold stored platelets in patients undergoing complex cardiothoracic surgery, but sufficient evidence based on clinical studies in routine use is still pending. The data about cryopreserved and lyophilized platelets are extremely promising, but further studies have to assess the efficacy and feasibility of these fascinating blood products. Several promising approaches are underway to find safe and effective alternatives to platelet transfusions, including auxiliary therapeutic strategies to support plasma coagulation, thrombopoietin mimetics, “Thromboerythrocytes”, “Plateletsomes”, “Infusible Platelet Membranes”, “Thrombosomes”, liposomal constructs, SynthoPlate^®^ (Haima Therapeutics, Cleveland, OH, USA) completely synthetic “platelets”, HAPPI (hemostatic agent via polymer peptide interfusion), and in vitro production of thrombocytes.

Altogether, several promising approaches are underway to find safe and effective improvements and/or alternatives to platelet transfusions. The reviewed issues are expected to have a major impact on current practices of platelet transfusion as soon as their efficacy and safety are revealed in current and upcoming clinical studies.

## Data Availability

Not applicable.
